# Clinical and economic impact of current ALK rearrangement testing in Spain compared with a hypothetical no-testing scenario

**DOI:** 10.1186/s12885-021-08407-1

**Published:** 2021-06-10

**Authors:** Ernest Nadal, Dolores Bautista, Luis Cabezón-Gutiérrez, Ana Laura Ortega, Héctor Torres, David Carcedo, Lucía Ruiz de Alda, J. Francisco Garcia, Paula Vieitez, Federico Rojo

**Affiliations:** 1 Catalan Institute of Oncology, Hospital Duran i Reynals, IDIBELL, L’Hospitalet de Llobregat, Spain; 2 Hospital Costa del Sol, Marbella, Spain; 3 Hospital Universitario de Torrejón, Torrejón De Ardoz, Spain; 4 Hospital Universitario de Jaen, Jaen, Spain; 5 Hospital Universitario Central de Asturias, Oviedo, Spain; 6 Hygeia Consulting S.A., Barcelona, Spain; 7 Roche Farma S.A., Madrid, Spain; 8 Hospital Universitario Fundacion Jimenez Diaz - CIBERONC, Madrid, Spain

## Abstract

**Background:**

Currently biomarkers play an essential role in diagnosis, treatment, and management of cancer. In non-small cell lung cancer (NSCLC) determination of biomarkers such as ALK, EGFR, ROS1 or PD-L1 is mandatory for an adequate treatment decision. The aim of this study is to determine the clinical and economic impact of current anaplastic lymphoma kinase testing scenario in Spain.

**Methods:**

A joint model, composed by decision-tree and Markov models, was developed to estimate the long-term health outcomes and costs of NSCLC patients, by comparing the current testing scenario for ALK in Spain vs a hypothetical no-testing. The current distribution of testing strategies for ALK determination and their sensitivity and specificity data were obtained from the literature. Treatment allocation based on the molecular testing result were defined by a panel of Spanish experts. To assess long-term effects of each treatment, 3-states Markov models were developed, where progression-free survival and overall survival curves were extrapolated using exponential models. Medical direct costs (expressed in €, 2019) were included. A lifetime horizon was used and a discount rate of 3% was applied for both costs and health effects. Several sensitivity analyses, both deterministic and probabilistic, were performed in order test the robustness of the analysis.

**Results:**

We estimated a target population of 7628 NSCLC patients, including those with non-squamous histology and those with squamous carcinomas who were never smokers. Over the lifetime horizon, the current ALK testing scenario produced additional 5060 and 3906 life-years and quality-adjusted life-years (QALY), respectively, compared with the no-testing scenario. Total direct costs were increased up to € 51,319,053 for testing scenario. The incremental cost-effectiveness ratio was 10,142 €/QALY. The sensitivity analyses carried out confirmed the robustness of the base-case results, being the treatment allocation and the test accuracy (sensitivity and specificity data) the key drivers of the model.

**Conclusions:**

ALK testing in advanced NSCLC patients, non-squamous and never-smoker squamous, provides more than 3000 QALYs in Spain over a lifetime horizon. Comparing this gain in health outcomes with the incremental costs, the resulting incremental cost-effectiveness ratio reinforces that testing non-squamous and never-smoker squamous NSCLC is a cost-effective strategy in Spain.

**Supplementary Information:**

The online version contains supplementary material available at 10.1186/s12885-021-08407-1.

## Background

Lung cancer is the leading cause of cancer death worldwide and was responsible for the highest number of deaths from cancer in Spain in 2018, with 22,133 deaths [1, 2]. Non-small cell lung cancer (NSCLC) accounts for 85–90% of lung cancer cases [3]. NSCLC has various histological subtypes, of which adenocarcinoma is the most common. In this subtype of NSCLC, several oncogenic and actionable drivers have been described, such as rearrangement of the anaplastic lymphoma kinase (*ALK)* gene, which is present in approximately 5% of cases of NSCLC [4]. Other actionable mutations have been detected in patients with NSCLC which affect the epidermal growth factor receptor (*EGFR*), *BRAF, ERBB2, MET* and gene fusions involving *ROS1, RET or NTRK1–3* [5]. About 70% of NSCLC patients have advanced disease at diagnosis, meaning they are not suitable for curative treatment [6]. The concept of personalized medicine came up with the identification of predictive biomarkers that enable the detection of patients who may obtain the greatest benefit with least associated toxicity from treatments such as chemotherapy or immunotherapy, or targeted therapy based on the individual tumor profile [7]. The detection of specific genomic alterations, such as *ALK* gene rearrangement in lung cancer patients, has evolved, leading to improved new detection techniques, and is currently considered indispensable in NSCLC for the prognostic evaluation, clinical decision-making and appropriate treatment [8]. Therefore, molecular genotyping at the diagnosis of advanced NSCLC is critical, since target identification is absolutely essential to allow access to therapies with the best efficacy and safety profile [9].

Currently, various methods allow the detection of *ALK* rearrangement in NSCLC, and the choice of assay depends on criteria such as personnel requirements, analytical parameters (sensitivity, specificity), associated costs, and others [10]. Molecular alterations in *ALK* may be identified by fluorescent in situ hybridization (FISH), immunohistochemistry (IHC), real-time polymerase chain reaction (RT-PCR), and new molecular techniques as next-generation sequencing (NGS) and NanoString nCounter [11, 12].

For some years, FISH has been the reference standard for detecting tumors carrying *ALK* rearrangement. However, growing evidence has shown that IHC testing is an efficient tool in the detection of *ALK* rearrangement, and confirmation of positive results by FISH is no longer needed [10, 13].

The Spanish Society of Medical Oncology (SEOM) and the Spanish Society of Pathology (SEAP) have published common recommendations to guide biomarker testing in patients with advanced NSCLC according to current evidence. *ALK* rearrangement should be tested in all patients with adenocarcinoma or non-squamous carcinoma, and also in squamous cell carcinoma in patients aged < 50 years of age and/or with low or no tobacco use. According to guidelines for molecular diagnosis in advanced NSCLC patients, molecular testing of *EGFR* and *BRAF* mutations*, ALK* and *ROS1* rearrangements and programmed death-ligand 1 (PD-L1) expression is considered mandatory [3, 14].

The main objective of this analysis was to quantify the clinical and economic impact of the current molecular testing scenario of NSCLC patients in Spain, focusing on the detection of *ALK* rearrangement, and comparing it with a hypothetical no-testing scenario.

## Methods

**Fig. 1:**
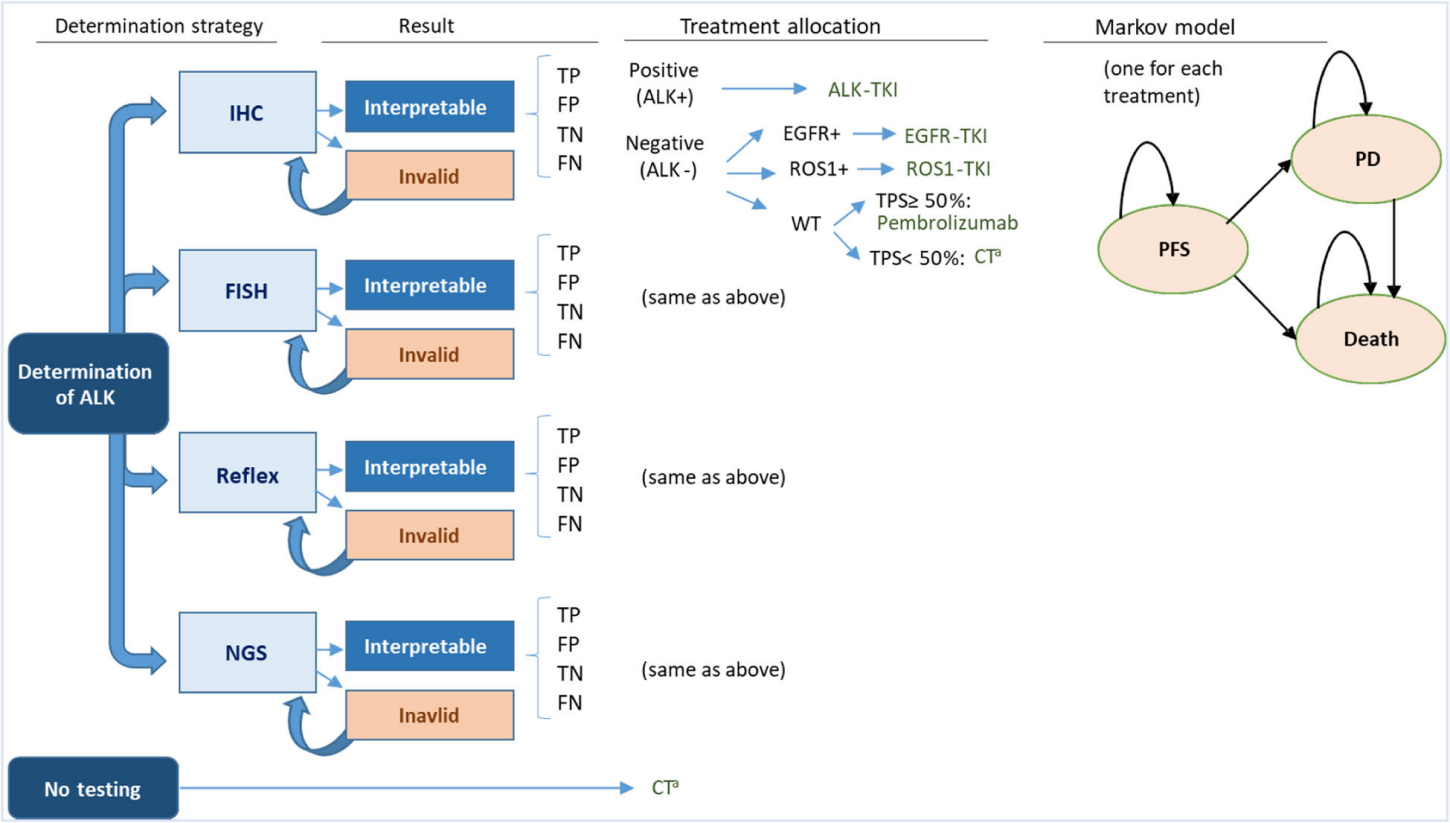
Model diagram. *ALK: anaplastic lymphoma kinase IHC: immunohistochemistry; FISH: fluorescent* in situ *hybridization; NGS: next-generation sequencing; TP: True positive; FP: false positive; TN: true negative; FN: false negative; EGFR:epidermal growth factor receptor; WT: wild-type; TPS: tumor proportion score TKI: tyrosine kinase inhibitors; PFS: progression-free survival PD: progressed-disease.* ^*a*^ Chemoterapy: cisplatin+pemetrexed; carbolplatin+paclitaxel+bevacizumab; cisplatin+pemetrexed+pembrolizumab

A joint model combining a decision-tree with Markov models was used to determine long-term health results and associated costs of the molecular testing scenario of NSCLC patients compared with a hypothetical no-testing scenario in Spain, using Microsoft Excel 2010 (Fig. 1).

The decision-tree model considered four alternative strategies for ALK rearrangement determination: IHC, FISH, IHC followed by confirmation of positive results by FISH (reflex), and NGS. If the result was invalid, the test was repeated. If the result was interpretable, the model determined four possible results: true positive (TP), false positive (FP), true negative (TN), and false negative (FN). These four possibilities reflect the typical outcomes of a binary diagnostic test (positive or negative), the underlying prevalence of the biomarker of interest (ALK+), and the sensitivity and specificity of the test strategies [15]:

**Table:** 

*-* *True positive*: *Prevalence* *sensitivity *-* *False positive*: (1–prevalence) * (1–specificity) *-* *True negative*: (1–prevalence) * specificity *-* *False negative*: *Prevalence* * *(1–sensitivity)*	

Based on the test results, a treatment was allocated and patients entered in the respective Markov model (in the no-testing scenario, patients enter directly into the non-targeted therapy Markov model), which was developed following an area under the curve structure with three health states: progression-free survival (PFS state), progressed-disease (PD state), and death state (absorbent state).

The Markov models considered monthly cycles, with a time horizon of 20 years (lifetime) and a discount rate of 3% for future costs and effects, as recommended by the guidelines for the evaluation of health technologies in Spain [16, 17].

The outcomes of the integrated model include life years (LY) and quality-adjusted life years (QALYs). Total costs (expressed in euros of 2020) were obtained from the health payer’s perspective, thus, direct health costs were considered in the analysis.

A multidisciplinary group of oncologists and pathologists, who are authors of this article, validated the included parameters and assumptions made, as well as the clinical feasibility of the results.

### Target population

A hypothetical cohort of patients with advanced or metastatic NSCLC, who were candidates for first-line treatment according to the molecular diagnosis, was included in the model. Following the current clinical guidelines for molecular diagnosis in lung cancer [14], both patients with non-squamous histology and those with squamous NSCLC who were never smokers were considered . Table 1 shows the estimation of the target population based on the literature [3, 18–22].

**Table 1:** Estimated target population

		%	Reference	N
1	Patients with lung cancer in 2020		(18)	28,475
2	Patients with NSCLC	85.0%	(3)	24,204
3	Stage VI in samples of NSCLC patients	54.5%	(19)	13,191
4	Patients with stage IV NSCLC non-squamous subtype	66.9%	(20)	8825
5	Patients with stage IV NSCLC squamous subtype	33.1%	(20)	4366
6	Patients with stage IV NSCLC squamous subtype, never smokers	16.0%	(21)	699
7	Candidate for ALK rearrangement diagnosis (steps 4 + 6)			9523
8	Patients finally tested for ALK (testing rate)	80.1%	(22)	**7628**

*NSCLC* Non-small cell lung cancer, *ALK* anaplastic lymphoma kinase

### Decision-tree parameters

Table 2 lists all inputs specific to the decision-tree model.

**Table 2:** Summary of decision-tree inputs

	Input	Reference
ALK determination strategies		
IHC	37.2%	[18]
FISH	11.6%	[18]
Reflex	46.5%	[18]
NGS	4.7%	[18]
Positivity rate in selected biomarkers		
ALK+	3.4%	[18]
EGFR+	13.6%	[18]
ROS1+	2%	[18]
PD-L1 ≥ 50%	33%	[18]
Percentage of invalid results		
With IHC or FISH	2.6%	[18]
With NGS	3.4%	[19]
Rebiopsy		
Probability of rebiopsy if invalid	77.1%	[20]
Specificity and sensitivity		
IHC	97.9% / 96.8%	[21]
FISH	99.8% / 90.9%	[22]
NGS	100%	Assumption^a^

*ALK* anaplastic lymphoma kinase, *IHC* immunohistochemistry, *FISH* fluorescent in situ hybridization, *NGS* next-generation sequencing, *EGFR* epidermal growth factor receptor

^a^*Considered the gold standard in the determination of ALK*

Current distribution in Spain of the four ALK determination strategies included in the model (Fig. 1) was obtained from the analysis of the LungPath database, as was the prevalence of positive results for the biomarkers of interest (positivity rate) [22].

In some cases, due to a lack of sample material or insufficient tumour cell percentage, it was not possible to accurately determine the test result and it was considered invalid, being a rebiopsy necessary sometimes. It was considered that in 77.1% of the invalid results a rebiopsy was carried (especially when patients were asymptomatic) waiting for results before starting treatment [24]. In the remaining 22.9%, chemotherapy was initiated without a molecular diagnosis result. It was assumed that the percentage of invalid results would be the same when the determination of ALK was made with IHC or FISH, but not with NGS, where the percentage of invalid tests may be higher [23].

The specificity and sensitivity parameters of each testing technique were obtained from Marchetti et al. for IHC [25] and for FISH [26]. The first article reports data from the European Quality Assessment in Italy, while the second analyzed 1031 samples using IHC, with FISH as the reference technique, and discrepancies analyzed by NGS. For NGS, 100% of specificity and sensitivity was assumed, as it is considered the gold standard in the determination of ALK.

The specific costs of the decision-tree were the costs of the tests used for molecular diagnosis and the cost of biopsy (applied when rebiopsy was required). The costs of the tests were agreed by the expert panel: € 60.28 for IHC; € 111.36 for FISH; € 475 for NGS; € 165 for the EGFR test; €100 for the ROS1 test; and € 130 for the PD-L1 test. For the biopsy, a cost of € 411.22 was considered [27].

Depending on the molecular testing result, a specific treatment was assigned (Fig. 1). Since the analysis is focused on ALK testing, when patients entered the Markov models, it was necessary to differentiate whether the positive result for ALK was a TP or a FP and whether the negative result was a TN or a FN. TP was differentiated from FP, as treatments targeting ALK (alectinib/crizotinib) are not effective in patients without ALK rearrangement, and therefore these treatments in patients who are FP for ALK provides almost no benefit. In the case of negative tests, it was assumed that in FN patients (who were ALK+ actually), the determination of EGFR and ROS1 would be negative, assuming that the concurrency of more than one mutation in ALK and EGFR or ROS1 is very infrequent [22].

### Markov model parameters

Eleven possible first-line treatments were considered, allocated according to the molecular diagnosis, entered the Markov models (one for each treatment) (Table 3). The distribution of treatments within the same molecular target was established by the panel of experts, reflecting current clinical practice in Spain.

**Table 3:** Distribution of treatments according to molecular diagnosis

Molecular diagnosis		Treatment	Distribution	
			BC	SA
ALK+		Alectinib	89.38%	80%
		Crizotinib	10.63%	20%
EGFR+		Erlotinib	15.0%	0.0%
		Gefitinib	30.0%	30.0%
		Afatinib	15.0%	5.0%
		Osimertinib	40.0%	65.0%
ROS1+		Crizotinib	100%	100%
WT	TPS 50%	Pembrolizumab	100%	100%
	TPS < 50%	Cisp + pmtrx	30.00%	40.0%
		Carb+ paclitx + bev	10.00%	5.0%
		Cisp + pmtrx + pembro	60.00%	55.0%

*ALK* anaplastic lymphoma kinase, *EGFR* epidermal growth factor receptor, *WT* wild-type, *TPS* tumor proportion score, *Cisp* cisplatin, *Carb* carboplatin, *pmtrx* pemetrexed, *paclitx* paclitaxel, *bev* bevacizumab, *BC* Base case, *SA* sensitivity analysis

Since the time horizon of the analysis was longer than the observation periods in clinical trials, it was necessary to extrapolate PFS and overall survival (OS) data, to obtain curves that allow to model transition between health states. In the absence of individualized patient data for each treatment, it was decided to use exponential models based on the median PFS and OS reported in the respective studies. In the recent update of the ALEX trial [28], used to obtain PFS and OS median for alectinib and crizotinib, median OS in the alectinib group was not reached, so extrapolation curves were obtained from the cost-effectiveness model of alectinib (data on file). Median PFS and OS for EGFR-targeted therapies were obtained from the FLAURA study [29, 30], and for ROS1-targeted therapies from PROFILE 1001 [31]. For wild-type (WT) patients treated with pembrolizumab in monotherapy, median PFS and OS were obtained from KEYNOTE-024 [32, 33], while from WT patients with tumor proportion score (TPS) < 50% median PFS and OS for the tree treatment strategies considered in the model were obtained from PARAMOUNT [34], Sandler et al. [35] and KEYNOTE-189 [36, 37].

Some adjustments had to be made in the PFS and OS exponential curves in the case of FP in ALK. The expert panel considered that most patients will have shown progression at the first follow-up visit and all of them will have progressed in the second visit. Based on this, a median of 2 months was assumed for the PFS in these patients, applying a stopping rule at 6 months (all patients progressed by then). Concerning OS, where there are more factors to consider, such as subsequent therapies, a median of 18 months was assumed.

The specific costs of the Markov models were the drug acquisition costs (both first-line and subsequent treatments) and the administration costs of intravenous treatments. Drug costs are expressed as the wholesale price considering the corresponding deductions according to Royal Decree-Law 08/2010 [38, 39]. For drugs where the dose depends on the patient characteristics, a mean body surface area of 1.81 and a mean weight of 72.885 kg was used [40]. Vial sharing for intravenous treatments was assumed.

Concerning intravenous administration costs, a cost of € 211 was considered (corresponding to day hospital visit) [27].

The model also included the cost of subsequent treatments (once patients progressed after first-line treatment and transit to the PD state). To avoid uncertainty regarding subsequent treatments and simplifying the model, only second-line treatments were considered. The percentage of patients receiving an active second-line treatment or who would receive best supportive care (BSC) was determined by the panel of experts, and as was the selection of the most representative second-line treatment (based on the first-line treatments received). The median PFS for second-line treatments was obtained from the literature [41–47]. All these parameters of the subsequent treatments are shown in an additional file [see Additional file 1].

The model incorporated utility values of 0.814 for PFS state, and 0.725 and 0.470 for the PD state with and without active treatment, respectively [48].

### Sensitivity analysis

To assess the uncertainty of some variables used in the analysis and to determine the robustness of the model and the results obtained, both deterministic and probabilistic sensitivity analyses (PSA) were performed.

#### Scenario analysis

Alternative scenarios to the base case were performed, modifying some assumptions, or exploring methodological alternatives.

- *Target population scenarios:* only patients with adenocarcinoma; and increasing the testing rate progressively (85% year 2 and 90% year 3).
- *Time horizon*: 10, 5 and 3 years, instead of lifetime (fixed at 20 years).
- *Decision-tree scenarios:* sequential rather than parallel diagnoses, and scenarios reducing invalid tests in IHC and FISH to 1% (not in NGS) and with 0% invalid tests in all options (IHC, FISH and NGS).
- *Markov-model scenarios:* alternative treatment allocation (Table 3), adjustments in the FP response for ALK (variations in PFS medians (1.5–3) and OS (15–21); and no stopping rule at 6 months), and without considering the cost of subsequent treatments.

#### Univariate analysis (one-way sensitivity analysis)

Some model variables were individually modified by 10% or 20% (depending on the uncertainty associated with the variable) with respect to the base case.

#### Bivariate analysis

Given the uncertainty associated with these variables, a specific analysis was performed where the sensitivity and specificity of IHC and FISH were simultaneously modified by ±1%, ± 2%, ± 3%, etc.

#### PSA

1000 simulations were performed using second-order Monte Carlo methodology, simultaneously modifying all model variables using a given distribution, in line with reported recommendations [49]. Utility values were modified using a beta distribution. For the body surface area and weight, the median PFS and OS, the positivity rate, the percentage of invalid results, and the probability of rebiopsy when results are invalid, a normal distribution was used. All unit costs were modified according to a gamma distribution.

## Results

**Fig. 2:**
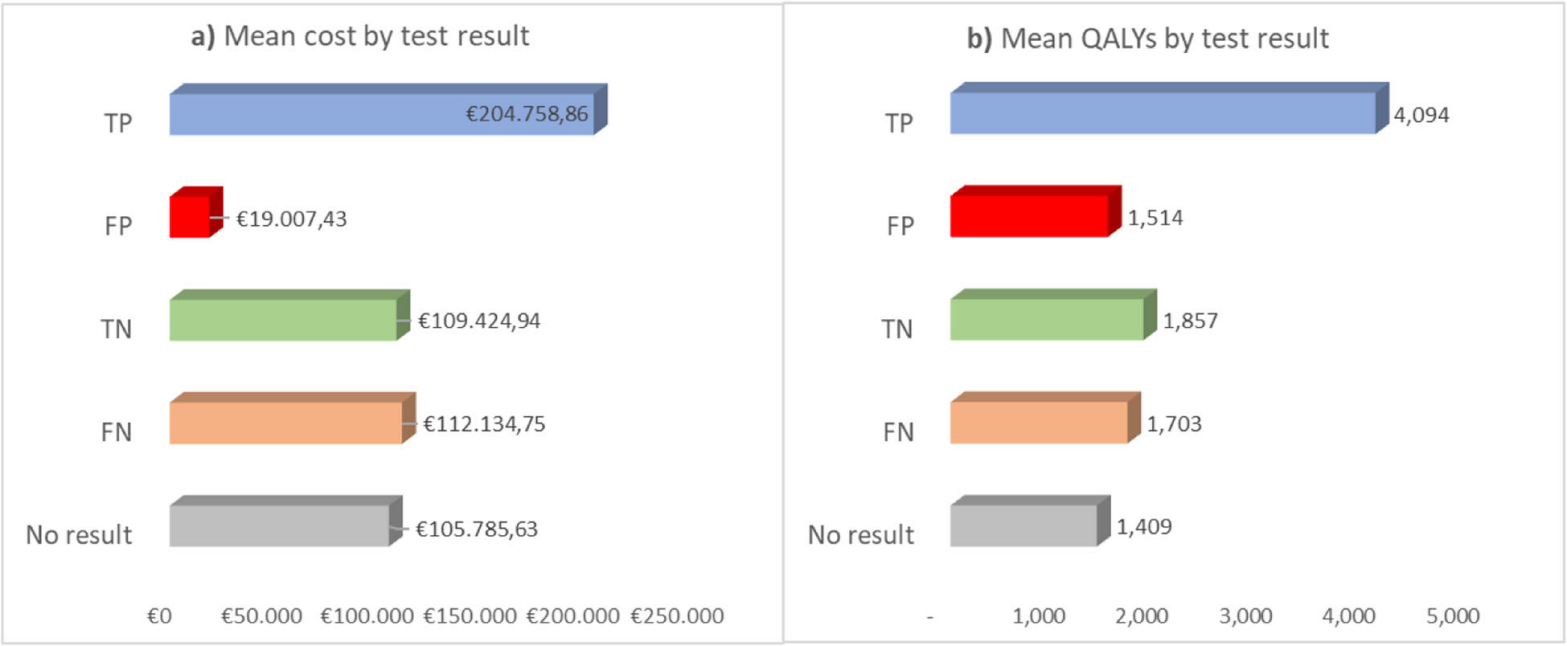
Mean results per patient. *TP: True positive; FP: false positive; TN: true negative; FN: false negative*

The costs and health outcomes obtained from the Markov models analyzed independently of the allocation between testing methods in the decision-tree, provided the mean cost as the mean QALYs per-patient according to the test result (Fig. 2). These results provide an assessment of costs and health outcomes per-patient, regardless of the technique used to obtain the TP, FP, TN and FN test outcome.

TP patients correctly treated with ALK-targeted therapies shown the best health outcomes, which also led to higher costs, as they spent more time in the PFS state while being treated with alectinib or crizotinib. FP patients had the worst health outcomes and the lowest cost, as they quickly progressed to second-line receiving few cycles of alectinib or crizotinib.

Table 4 lists the results of the base case, showing the total health outcomes and costs for the current testing scenario and the non-testing scenario, including the incremental results comparing both scenarios.

**Table 4:** Base case results

	Current scenario tested	No-testing	Difference
*Cost of testing*	*€ 3,613,701*	*€ 0*	*€ + 3, 613, 701*
*Cost of treatment*	*€ 854,664,411*	*€ 806,959,058*	*€ + 47, 705, 353*
Total costs	**€ 858,278,111**	**€ 806,959,058**	**€ + 51, 319, 053**
LY	21.233,59	16.173,45	+ 5060.14
QALYs	14.654,68	10.748,05	+ 3906.64
ICER (€/LY gained)			**€ 10,142/LY**
ICUR (€/QALY gained)			**€ 13,136/QALY**

*LY* life years, *QALY* quality-adjusted life years, *ICER* incremental cost-effectiveness ratio, *ICUR* incremental cost-utility ratio

The current strategy of testing patients with advanced NSCLC provided a gain of 3907 QALYs compared with the no-testing scenario, over a 20-year time horizon. This also entailed higher costs, including those of the tests themselves, but mainly due to the cost of targeted treatments. The comparison of costs and health outcomes through the incremental cost-effectiveness ratio (ICER), shows that the current ALK testing strategy in Spain is cost-effective, as it was below the cost-effectiveness thresholds commonly considered in Spain [50, 51].

### Sensitivity analysis

The target population scenarios affect only the absolute value of the results (in both scenarios compared), maintaining the ICER constant. If only patients with adenocarcinoma were tested, only 3620.06 QALYs would be gained (287 fewer QALYs gained than in the base case). In the scenario where the testing rate was increased up to 90% after 2 years, the gain in QALYs compared with the non-testing scenario would be 4389.48 QALYs (483 more than in the base case).

Shorting time horizon up to 10, 5 and 3 years, increased the ICER to € 14,223, €16,360, and € 17,275/QALY, respectively.

The decision-tree scenarios had a slight influence on the results, and only affected the current testing scenario (the non-testing scenario remained the same). A sequential instead of a parallel diagnosis slightly decreased the costs of the current testing scenario (€ -391,375), and the ICER was reduced to € 13,036/QALY. Reducing the percentage of invalid tests also hardly impacted on the ICER (€ 13,139 and € 13,140 /QALY for the two scenarios posed, respectively).

The Markov model scenarios had the greatest influence on the base case results. When the allocation of treatments shown in Table 3 was modified, the ICER increased to € 24,660/QALY, mainly due to the total cost for a lifetime horizon of the non-testing scenario being reduced from € 806,959,058 to € 749, 855,143 (€-57,103,915), while in the current testing scenario it was only reduced by € 6,911,633. The adjustments in the response to FP for ALK only affected the current testing scenario, although the changes respect to the base case were minimal (± 0.01% with respect to costs and QALYs). The ICER resulting from modifying the PFS and OS in FP patients, or not considering the 6-months stopping rule were € 13,134, €13,137, and € 13,148/QALY, respectively. Not considering second-line treatments reduced the ICER to € 10,059/QALY, due to a reduction of € 58,666,678 and € 46,645,461 in the total costs of the current and non-testing scenarios, respectively.

**Fig. 3:**
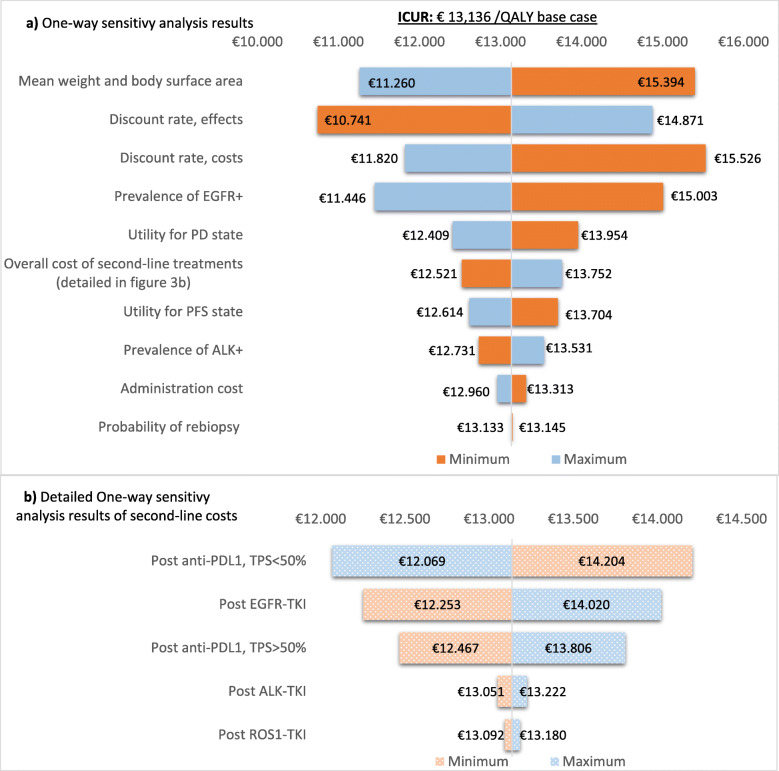
Tornado diagrams. *ICUR: incremental cost-utility ratio*

The results of the univariate analysis, where the impact of the extreme values of each variable on the base case incremental cost-utility ratio (ICUR) and are assessed, are represented by a tornado diagram in Fig. 3. Patient characteristics (mean weight and body surface area), discount rate (for both cost and effects), and prevalence of EGFR+ shown the greatest impact on base case results (Fig. 3a). The tornado diagram represented in Fig. 3b detailed the analysis of the second-line costs.

In the bivariate analysis, the specificity and sensitivity base case parameters are increased and decreased simultaneously, and Table 5 shows how the 3907 QALYs gained in the base case are modified accordingly. The gradient from green to red shows the difference from the base case.

**Table 5:**
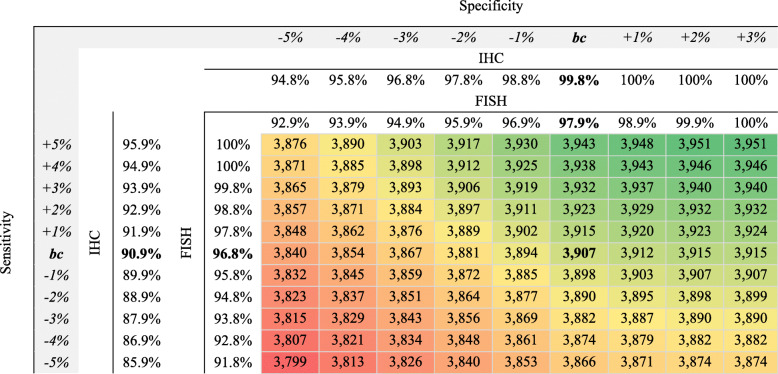
Bivariate analysis

*BC* Base case, *IHC* immunohistochemistry, *FISH* fluorescent in situ hybridizatio

**Fig. 4:**
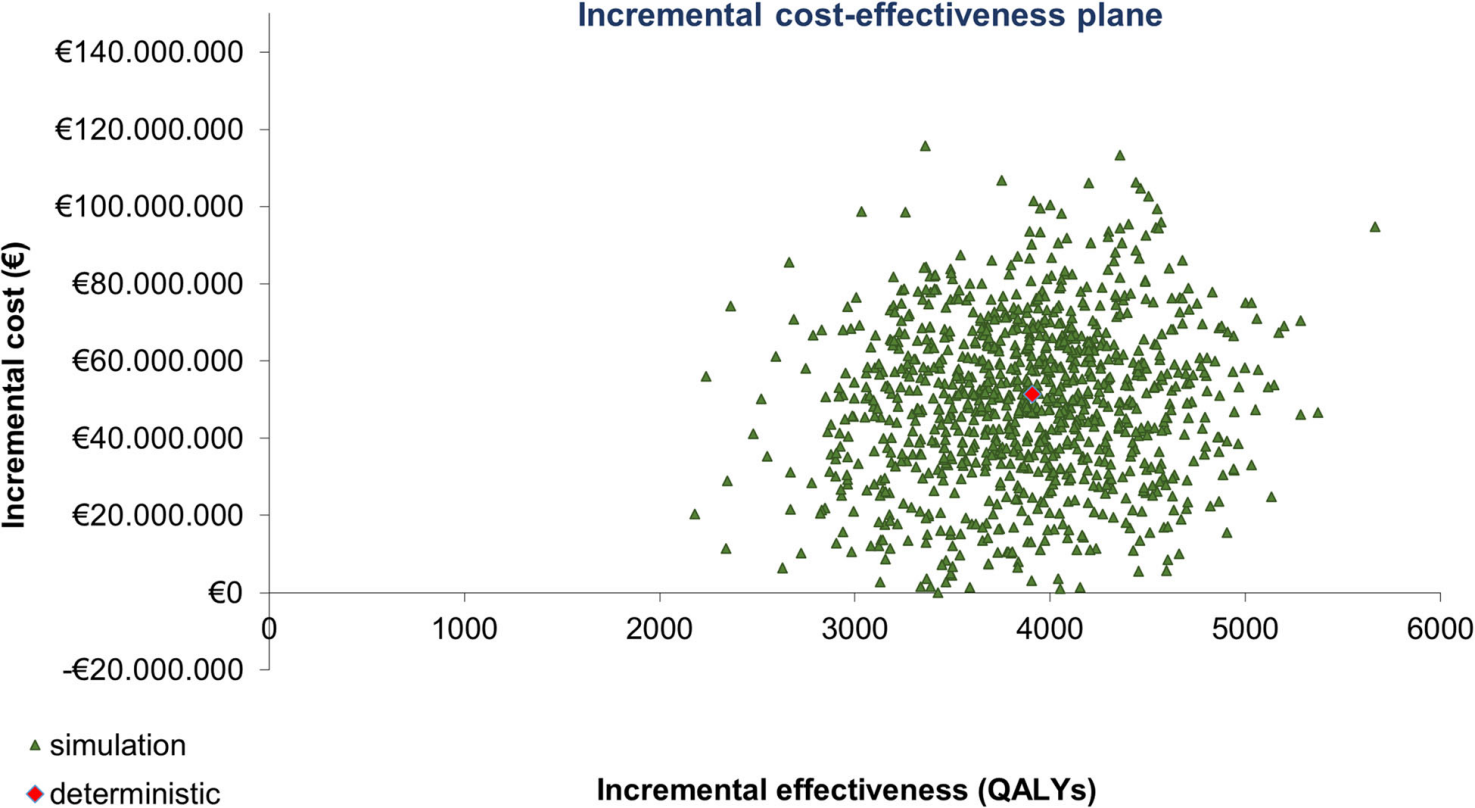
PSA results. Incremental cost-effectiveness plane. *QALY: quality-adjusted life years*

Figure 4 shows the PSA results represented by an incremental cost-effectiveness plane. The means obtained from the 1000 simulations (€ + 51,319,053 and 3907 QALYs gained with respect to the non-testing scenario) are in line with the deterministic results in Table 4.

## Discussion

In recent years there have been multiple changes in the approach to lung cancer treatment and, in particular, to NSCLC, including the identification of biomarkers that allow treatment selection in some subgroups of patients with advanced disease [52]. Deciding which biomarkers and which subgroup of patients should be tested is clinically relevant, and it must be assessed at diagnosis, since the first treatment a cancer patient receives should be the most effective according to their clinical and molecular features [52].

Specifically, the detection of *ALK* rearrangements in patients with NSCLC provides unquestionable clinical benefits. A systematic review and meta-analysis by Elliott et al. [53] concluded that, ALK inhibitors improved PFS relative to chemotherapy. Also, treatment-related deaths were found to be infrequent among ALK-positive NSCLC patients [53]. Molecular testing for these rearrangements (along with molecular alterations in other biomarkers) is considered mandatory in all patients with non-squamous NSCLC or patients with squamous NSCLC younger than 50 years or with low tobacco exposure, according to the current clinical guidelines [14, 54, 55]. Next-generation ALK tyrosine kinase inhibitors (TKI) have generally replaced crizotinib, the first-generation TKI, as front-line treatments for patients with ALK-positive NSCLC [28]. Alectinib was associated with longer PFS and lower toxicity than crizotinib and showed activity against central nervous system (CNS) disease in patients with ALK-positive NSCLC [56].

Pathology departments must work in coordination with the other services involved in the diagnosis and treatment of NSCLC patients and are key to optimizing the available resources and the clinical management of these patients [52]. Therefore, to evaluate the efficiency of the management of patients with advanced NSCLC in Spain, a comprehensive approach to molecular diagnosis in conjunction with pharmacological treatment is essential.

Our study is the first one that uses this comprehensive approach to assess the long-term clinical and economic impact of the current molecular testing scenario in Spain. The Markov models allow the calculation of long-term costs, LYs, and QALYs for each treatment that is allocated based on the molecular result, and together with the positivity rate of each biomarker, the mean cost as the mean QALYs per patient were calculated. The TP patients provide, by far, the best health outcomes while the FP represented the worst health outcomes, therefore specificity and sensitivity have great importance. Bivariate analysis showed that when specificity and sensitivity were decreased equally (in the same percentage), gained QALYs were slightly lower with the decreased specificity than with the decreased sensitivity. Complementing the Markov models, the decision tree model allows to compare between the current molecular testing scenario (considering 4 alternative strategies for *ALK* rearrangement determination, IHC, FISH, reflex, and NGS) and the no-testing scenario. The ICER obtained (both base case and sensitive analysis) confirms that determining *ALK* rearrangement in patients with advanced NSCLC is a cost-effective strategy, since it is below the cost-effectiveness thresholds usually considered in Spain [50, 51]. Therefore, our findings support and confirm the recommendations of national guidelines [14]. Preliminary results of our study were presented in ISPOR 2020 in the form of poster [57] and 2020 World Conference on Lung Cancer (WCLC) through an oral communication” [58].

The described comprehensive approach has also been used in other economic assessments. Lee et al. published a generalized model that allows the evaluation of molecular diagnosis and subsequent targeted therapy [59]. Using this generalized model, the same author determined the cost-benefit ratio of ALK testing using different techniques for subsequent targeted treatment with crizotinib. The authors concluded that more than 95% of the total cost corresponded to the treatment and, therefore, the model was particularly sensitive to the price or clinical benefits of crizotinib and the accuracy of the diagnostic test, but was not particularly impacted by the cost of the diagnostic test [59].

Other studies have been based on similar approaches to compare *ALK* rearrangement detection techniques. Doshi et al. [10] concluded that both in the United States (U.S.) and Europe, *ALK* testing only by IHC is the lower-cost strategy compared with using only FISH, reflex testing, or performing both tests in parallel. Paolini et al. [13] assessed the clinical and economic benefits of using IHC for *ALK* rearrangement testing in Italy, and concluded that increased use of D5F3 would provide savings of € 468,000, due to its lower acquisition cost compared with FISH and a comparable detection rate. Lu et al. [60] evaluate the cost-effectiveness of the ALK tests followed by crizotinib compared to the standard chemotherapy in advanced NSCLC from the Chinese healthcare system perspective. They found that two testing methods for ALK assessed (NGS and multiplex PCR) followed by subsequent crizotinib treatment, are both cost-effective compared to standard chemotherapy without ALK testing [60]. Also, two recent analyses have focused on the cost-effectiveness of using NGS in NSCLC patients, finding that this technology would be cost-effective compared to single testing [61, 62].

Unlike these economic assessments, our analysis is not intended to compare diagnostic tests, the aim is to analyze the current scenario of molecular testing in Spain for patients with NSCLC, quantify the costs and long-term health outcomes, and compare them with a hypothetical non-testing scenario.

The study with the most similar objective was performed by Loubiére et al. [63], although the methodology differs entirely, as it prospectively evaluated a cohort of 843 patients in 19 French hospitals, while our analysis is based on a theoretical pharmacoeconomic model. Interestingly, their results were similar to our findings, since they reported that the “at least one biomarker status known” strategy is cost-effective compared with the “no biomarker testing” strategy, with an ICER of € 13,230 per QALY gained, while our base case ICER was 13,136 per QALY gained [63].

Our study has some limitations. First, the limitations inherent to pharmacoeconomic models, which are characterized by some degree of structural rigidity which cannot completely recapitulate the complexity of the clinical practice. For example, reproducing the pathological and molecular diagnosis process accurately is complicated, due to the complexity of the process and the high variability of the studies identified. Therefore, to simplify the model we did not include the pre-analytic phases in our study. Although turnaround times are a key factor in initiating treatment at an appropriate time, no evidence has been found regarding their influence on the treatment effectiveness, and therefore the time from diagnosis to initiation of treatment was not included as a variable in our model. As stated in the literature and confirmed by the expert panel, turnaround times may vary from 2 to 5 [10] to 6–12 working days [64], with significant variations between centers (diagnostic outsourcing may add 1 week to the turnaround time according to the experts). A study designed to establish whether reducing the turnaround time could reduce the healthcare resources utilization and could improve the results obtained in this analysis would be of interest.

Secondly, in the molecular diagnosis phase, the percentage of invalid results and the specificity and sensitivity parameters of each technique were obtained from the literature, in the absence of a specifically designed study that could provide these data together. The definition of an invalid result may vary between studies and rely on the reference technique used to calculate the specificity and sensitivity. The studies published by Marchetti et al. [25, 26] were selected by the expert panel, since based on these parameters of specificity and sensitivity, the model predicted similar results to those observed in current clinical practice. Similarly, although the experts agreed that 2.6% of the invalid tests reported in LungPath study would apply equally to IHC and FISH, usual practice shows significant variations according to the histology and between reference and smaller centers, where pre-analytical conditions may lead to a higher failure rate when using FISH. For NGS, a higher percentage of invalid results has been reported, as it is a newer technique and is of greater difficulty in the pre-analytic phase, and is more likely that samples will not have enough quantity or quality for NGS testing.

In addition, the percentage of invalid results could also be reduced in the future through the increased use of liquid biopsies. Liquid biopsy is a novel powerful tool for tumor genotyping and multiple levels of evidence support its use as an alternative strategy for molecular testing in advanced NSCLC, especially when tumor tissue is not sufficient [65].

Thirdly, the clinical management of patients with NSCLC reproduced by the Markov models also has some limitations. A lifetime horizon was adopted, as this made it possible to capture all the benefits of the targeted therapies in terms of LY and QALYs gained, in line with the economic evaluation developed by Lee et al. [57] which was based on the cost-effectiveness analysis of crizotinib. However, sensitivity analyses of the time horizon showed that determining *ALK* rearrangement remained cost-effective as compared with the non-testing strategy. Due to the lack of individualized patient data for each treatment, the utilization of exponential parametric models (adjusted according to the median PFS and OS) was assumed. This assumption, validated by the panel of experts, is in line with published economic assessments of alectinib [48, 66], where the exponential models showed the best fit to the ALEX study data. Since our analysis focused on molecular testing of patients with advanced NSCLC and the consequent first-line of treatment, the subsequent lines (second-line) only show the influence in economic terms, were introduced in the model as a one-off cost.

Fourthly, concerning costs, it is difficult to set the real price of diagnostic tests, since there are wide variations between centers, and in many cases, the cost is borne by research funds or by pharmaceutical companies since they are not reimbursed by the Spanish National Health System. Besides, as the analysis focused on the determination of *ALK*, not all the benefits of using NGS, which allows to evaluate simultaneously multiple biomarkers instead of testing each biomarker one at a time, cannot be captured. An economic assessment of the use of NGS instead of testing individually the main biomarkers will require a specifically designed model and is beyond the scope of this work.

To overcome or minimize these limitations and the associated uncertainty, we performed sensitivity analyses that confirmed the robustness of the results obtained. In addition, all assumptions made, the parameters considered, and the results obtained were validated by the panel of experts.

## Conclusions

In conclusion, our analysis shows that the detection of *ALK* rearrangements in patients with NSCLC in Spain provides important clinical benefits compared with not testing in these patients. Appropriate *ALK* testing yields about 4000 QALYs gained over a 20-year time horizon. When these results are compared with the incremental cost, the resulting ICER shows that the current molecular testing strategy in Spain not only increases and improves the lives of patients with NSCLC but is also cost-effective for the Spanish National Health System.

## Supplementary Information

**Additional file 1.** Definition of subsequent (second-line) treatments.

## Data Availability

Qualified researchers may request access to individual patient level data through the clinical study data request platform (https://vivli.org/). Further details on Roche’s criteria for eligible studies are available here (https://vivli.org/members/ourmembers/). For further details on Roche’s Global Policy on the Sharing of Clinical Information and how to request access to related clinical study documents, see here (https://www.roche.com/research_and_development/who_we_are_how_we_work/clinical_trials/our_commitment_to_data_sharing.htm).

## Abbreviations

NSCLC: Non-small cell lung cancer
ALK: Anaplastic lymphoma kinase
EGFR: Epidermal growth factor receptor
FISH: Fluorescent in situ hybridization
IHC: Immunohistochemistry
RT-PCR: Real-time polymerase chain reaction
NGS: Next-generation sequencing
SEOM: Spanish Society of Medical Oncology
SEAP: Spanish Society of Pathology
PD-L1: Programmed death-ligand 1
TP: True positive
FP: False positive
TN: True negative
FN: False negative
LY: Life years
QALY: Quality-adjusted life years
PFS: Progression-free survival
OS: Overall survival
ICER: Incremental cost-effectiveness ratio
ICUR: Incremental cost-utility ratio
