# Supplementary material for: Clinical and economic impact of current ALK rearrangement testing in Spain compared with a hypothetical no-testing scenario
Source: BMC Cancer. 2021 Jun 10;21:689. doi: 10.1186/s12885-021-08407-1 (PMC8194132; doi:10.1186/s12885-021-08407-1)
Supplement: Supplementary file 1 — Additional file 1. Definition of subsequent (second-line) treatments. [file 12885_2021_8407_MOESM1_ESM.pdf]

Additional file 1. Definition of subsequent (second-line) treatments

Molecular diagnosis		First-line treatment		Second-line treatment				
				Active treatment			Non-treatment	
				%	Treatment		%	Treatment
ALK+		Alectinib		*80,00%*	*Cisp + pmtrx*		*20,00%*	*BSC*
		Crizotinib		*80,00%*	*alectinib*		*20,00%*	*BSC*
EGFR+		Erlotinib		*70,00%*	*osimertinib*		*30,00%*	*BSC*
		Gefitinib		*70,00%*	*osimertinib*		*30,00%*	*BSC*
		Afatinib		*70,00%*	*osimertinib*		*30,00%*	*BSC*
		Osimertinib		*70,00%*	*Cisp + pmtrx*		*30,00%*	*BSC*
ROS1+		Crizotinib		*80,00%*	*Cisp + pmtrx*		*20,00%*	*BSC*
WT	***TPS≥50%***	Pembrolizumab		*60,80%*	*Cisp + pmtrx*		*39,20%*	*BSC*
	TPS<50%	Cisp + pmtrx		*46,60%*	*Inmunotherapies[a] (75%); docetaxel + nintedanib (25%)*		*53,40%*	*BSC*
		Carb+ paclitx + bev		*46,60%*			*53,40%*	*BSC*
		Cisp + pmtrx + pembro		*30,50%*	*docetaxel + nintedanib*		*69,50%*	*BSC*

ALK: anaplastic lymphoma kinase; EGFR: epidermal growth factor receptor; WT: wild-type; TPS: tumor proportion score; Cisp: cisplatin; Carb: carboplatin; pmtrx: pemetrexed; paclitx; paclitaxel; bev: bevacizumab; BSC: best supportive care.

[a] *Atezolizumab, pembrolizumab and nivolumab*
